# Judging the emotional states of customer service staff in the workplace: A multimodal dataset analysis

**DOI:** 10.3389/fpsyg.2022.1001885

**Published:** 2022-11-11

**Authors:** Ping Liu, Yi Zhang, Ziyue Xiong, Yijie Wang, Linbo Qing

**Affiliations:** ^1^School of Business, Sichuan University, Chengdu, China; ^2^School of Business and Tourism Management, Yunnan University, Kunming, China; ^3^School of Electronic and Information Engineering, Sichuan University, Chengdu, China

**Keywords:** workplace emotions, emotion recognition, customer service staff, picture-type scale, multimodal dataset

## Abstract

**Background:**

Emotions play a decisive and central role in the workplace, especially in the service-oriented enterprises. Due to the highly participatory and interactive nature of the service process, employees’ emotions are usually highly volatile during the service delivery process, which can have a negative impact on business performance. Therefore, it is important to effectively judge the emotional states of customer service staff.

**Methods:**

We collected data on real-life work situations of call center employees in a large company. Three consecutive studies were conducted: first, the emotional states of 29 customer service staff were videotaped by wide-angle cameras. In Study 1, we constructed scoring criteria and auxiliary tools of picture-type scales through a free association test. In Study 2, two groups of experts were invited to evaluate the emotional states of customer service staff. In Study 3, based on the results in Study 2 and a multimodal emotional recognition method, a multimodal dataset was constructed to explore how each modality conveys the emotions of customer service staff in workplace.

**Results:**

Through the scoring by 2 groups of experts and 1 group of volunteers, we first developed a set of scoring criteria and picture-type scales with the combination of SAM scale for judging the emotional state of customer service staff. Then we constructed 99 (out of 297) sets of stable multimodal emotion datasets. Based on the comparison among the datasets, we found that voice conveys emotional valence in the workplace more significantly, and that facial expressions have more prominant connection with emotional arousal.

**Conclusion:**

Theoretically, this study enriches the way in which emotion data is collected and can provide a basis for the subsequent development of multimodal emotional datasets. Practically, it can provide guidance for the effective judgment of employee emotions in the workplace.

## Introduction

The attributes of emotion, such as intangibility, high intensity, and contagiousness, highlight the importance of recognizing and managing employees’ emotions in the workplace (Camacho et al., 1991; Liu et al., 2019). Workplace emotion refers to a subjective experience that comes from an individual’s physiological arousal evoked by workplace stimuli (Jordi et al., 2015; Rueff-Lopes et al., 2015; Liu et al., 2019). A recent IMF’s World Trade Statistical Review (July 2021) projected that the worldwide service trade had dropped by 16% in 2020 while online services had risen by 9% in 2021, with even greater growth expected in some countries. Given the high-interaction and high-participation nature of service industries (Zemke and Bell, 1990; Rueff-Lopes et al., 2015; Liu et al., 2019), service staff are usually required to manage their emotions, and only demonstrate those emotions allowed by organizational policies. This phenomenon is often interpreted as emotional labor (Hochschild, 1979; Hatfield et al., 1993; Grandey and Melloy, 2017). Providing services that evoke emotional labor can increase employees’ workload (Destephe et al., 2015), aggravate work pressure, and cause job burnout (Baeriswy et al., 2021; Schabram and Tseheng, 2022), ultimately leading to emotional disorders (Farchione et al., 2012) and poor enterprise performance (Rueff-Lopes et al., 2015; Liu et al., 2019). Therefore, it is of vital importance for enterprises, especially those in the service industry, to acknowledge and effectively manage emotions in the workplace.

In the modern service industry, the ways of delivering service to customers have changed (Jeremy et al., 2005; Liu et al., 2019). Voice-to-voice communication has gradually become a prevailing method for service enterprises to attend to customers’ needs (Rueff-Lopes et al., 2015; Sparks et al., 2015). Meanwhile, the application of 5G and other digital technology is enriching the ways of online services, including online healthcare, online shopping, and telecommuting (Khan and Zhang, 2007; He et al., 2013). Under these circumstances, voice-to-voice interaction between service employees and customers has become a key element of service delivery (Barry and Crant, 2000; Goldberg and Grandey, 2007; Rueff-Lopes et al., 2015). Some scholars have found that negative externalities, such as business bankruptcies and prolonged isolation (Bherwani et al., 2020), will led to rising stress and workloads (Wheaton et al., 2021). Thus, in the process of providing service, employees are more likely to be susceptible to negative emotions, which threatens the results of the voice-to-voice service delivery. Therefore, judging the employees’ emotional states in the workplace, it is of great practical significance for managers.

To date, although there has been an increase in studies of employees’ emotions, there are shortcomings in the literature. First, in terms of the research methods, to achieve statistics on individual emotions, most projects adopt the paradigms of case studies applying stimulus materials (Michel, 2001; Du and Fan, 2007) or self-report scales, such as PANAS, PAD, SDS, etc. (Bradley et al., 2001; Li et al., 2005; Kuesten et al., 2014). While these approaches are reasonable, they overlook the influence of situational and contextual elements on individual emotions to some extent. Further, it is hard to avoid common method bias with case stimulus and questionnaires (Jordan and Troth, 2019). Second, most of the research results are presented in the form of discrete emotions and thereby lack the characteristics of real situations (Ekman, 1994; Gao et al., 2019). Because individual emotions at a specific point in time are a complex combination of discrete emotions, the practical guidance offered by existing studies is relatively weak. Third, some research groups have come to realize that single-modal emotion measurement cannot accurately identify the individual emotional state, and that emotion recognition needs to be treated as a multi-modal problem in the research field of OB (organization behavior) and psychology (Balconi and Fronda, 2021; Guedes et al., 2022; Zhao et al., 2022). This is because human emotions are relatively rich and complex in terms of expression (Lackovi, 2018; Liu et al., 2019). For example, a sentence may contain multiple, even conflicting emotions; a positively worded sentence, for instance, may express sarcasm (Gao et al., 2019). Therefore, it is necessary to analyze the emotional states of employees in workplaces from a multi-modal perspective, so as to enrich the methods of organization behavior and psychological research.

This study aims to find out which emotional modality can most accurately convey the emotions of customer service staff in the workplace and how to do so. Based on the multimodal emotion recognition method (Lahat et al., 2015; Baltrušaitis et al., 2018; Ethriaj and Isaac, 2019), emotions can be divided into three fundamental modalities: body language, voice, and facial expression. This multimodal classification features both visual and auditory channels of an individual’s physiology (Wang et al., 2019). Although data of each modality can convey the emotional state of customer service in a workplace, in this article we mainly focus on comparing the differences of modalities that have a high level of practical relevance. The innovations of this study are as follows. First, from the perspective of situational embedding, the research team observed the work emotions of customer service in a real service-oriented enterprise. Second, in terms of the experimental research paradigm, this paper constructed a multimodal data set, compared the heterogeneity of different emotional modalities, and extracted the key elements of the emotional states of customer services employees. Third, this paper summarized the theoretical and practical significance of managing service employees’ emotional states and suggested future directions for this research filed.

## Theoretical background

### Workplace emotions

Emotions usually reflect people’s attitudes toward objective things or situations (Stanger et al., 2017). Emotions are short-lived and high-intense responses that develop automatically when an organism is stimulated by an external irritant (Venkatraman et al., 2017). In academia, there are several different views on the understanding of emotion. The biological view holds that emotions arise from the nervous system, and they are a product of evolution of living creatures (Sun et al., 2016). The functionalist perspective believes that emotions evaluate a particular environment, and are specific mental activities produced by the individual in response to stimuli from personally meaningful events (Boiten et al., 1994; Barret, 1998; Stanger et al., 2016). Campos et al. (1989) defined emotion as “not just feelings, but rather the process of maintaining, disrupting or maintaining the relationship between an organism and environment, when such relationship has implication to the person.” The organizational perspective holds that emotions are a kind of “imitation-response” mechanism, which is generated by individual’s interaction with their environment (Sroufe, 1996; Lewis, 1998). The socio-cultural perspective holds that emotions are a profound psychological and physical experience, mediated by a variety of social and cultural factors (Zhang and Lu, 2013).

At present, the interpretation of emotion continues to be debated in the academic community, but there are several common features. First, emotion is a physiological and psychological state, including subjective experience, behavioral expressions, and peripheral physiological responses (Nitsche et al., 2012). Second, emotion is typically triggered by a specific reason or situation with short duration and high intensity (Camacho et al., 1991; Ekman, 1994; Gao et al., 2019). Third, emotion is responsive to the external environment and manifests as a form of experience (Perveen et al., 2020). Fourth, emotion has two attributes: biological and cultural. Individuals who grow up in different cultures may have different ways of expressing the complexity of emotions.

In the context of this research, workplace emotion in this article refers to an emotional experience that is felt by customer service staff during the service process. It is a physiological response to internal and external stimuli, with the characteristics of short duration and high intensity.

### Multimodal emotion recognition method

Emotion recognition is a dynamic process that aims to identify the emotional states of individuals (Jess et al., 2020). The classification of emotions falls into two schools of thought: categorical and dimensional. The categorical approach views that emotions can be summarized in terms of basic emotions such as joy, anger, sadness, fear etc. (Ekman, 1994). However, the dimensional approach argues that people often have difficulty when evaluating, distinguishing, and describing their emotions. That is to say, emotions are more like a blurred set of conceptions that blend with each other than a discrete system. Therefore, the theory of dimensional emotion has been favored in academic circles. After in-deep study of pleasure-arousal-dominance (PAD) (Mehrabian and Russell, 1974). Russell (1980) pointed out a structure model of affective experience, he believed that various emotions were not separate categories, but have certain values in the two dimensions of valence (pleasure) and arousal (alertness). So, valence is a unpleasant-pleasant experience, a process from one extreme to the origin of coordinates and then to the other extreme (e.g., from distress to ecstasy), and the arousal is the feeling of vitality or energy, such as the progression from drowsiness, relaxation and alertness to excitement (Russell, 1980; Waston and Tellegen, 1985). Therefore, according to the level of emotional experience, and the degree of energy and vitality respectively, we divided the emotional valence and arousal into three levels, namely, valence (negative, neutral, positive), arousal (low, medium, high).

Modality is a representable, objective social symbol system that is an important vehicle for emotional signaling (Ethriaj and Isaac, 2019; Gao et al., 2019). There are two major views on the interpretation of modality. One sees modality as the form of data representation, in which text, video, image, and sound are separate modalities (Hong and Tam, 2006; Baltrušaitis et al., 2018; Balconi and Fronda, 2021). The other views modality as the mechanism of data collection, whether through self-report scales or electrophysiological equipment (Wang et al., 2022). Multimodal emotional recognition is more accurate than traditional single-modal emotional recognition, because information if integrated from different modalities (Lahat et al., 2015; Yoon et al., 2015; Wang et al., 2019). Scholars studying emotions have found that single-modal data of emotion are prone to greater errors in emotional recognition than multi-modal data. For instance, Battaglia (2010) and Lambercht et al. (2012) found that there were significant errors in recognizing the facial expressions of anger and fear in subjects of different ages, which suggests that single-modal emotion recognition results are susceptible to the influence of the subject’s age. Aviezer et al. (2012) and Wang et al. (2021) researched online images and constructed a mixed stimulation material (including limbs, expressions, and gestures). Their results consistently show that limbs are more accurately convey the tennis players’ emotional statues. Therefore, compared to multimodal emotion recognition, we propose that single-model recognition is susceptible to subjective and objective factors, resulting in distorted judgment.

In summary, multimodal data sets have a diversity of data representations (such as visual and auditory) and are collected in at least two different channels (such as self-report scale or electrophysiological equipment). This study constructs a multimodal data set to explore the differences of each modality (body language, voice, and facial expression) in conveying the emotions of customer service staff. Further, this study provides recommendations for effectively identifying and managing customer service sentiment in the workplace.

## Overview of the study

**FIGURE 1:**
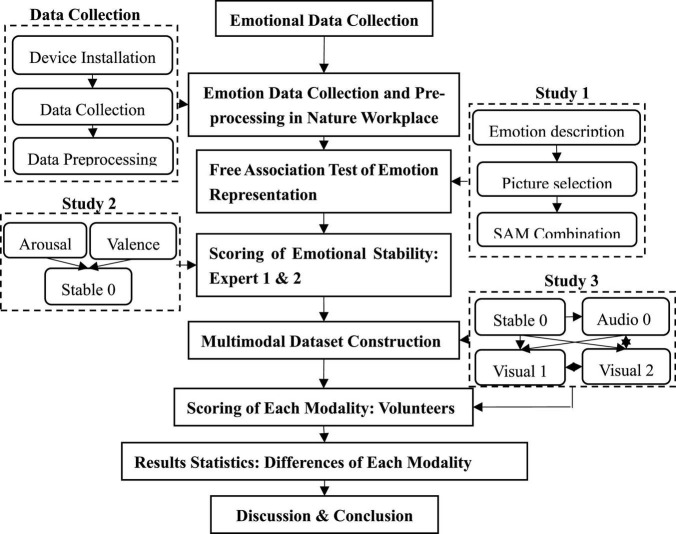
Research framework.

Our research follows an experimental research approach. We collected the emotional data of 29 customer service staff from March 15 to March 30, 2021. Three distinct sample groups were recruited to evaluate the emotions of the staff (Expert 1: doctors in organizational behavior and psychology; Expert 2: doctors in computer image emotion recognition; Volunteers: a social group recruited online). The research framework of this study is shown in Figure 1.

This study was field at the Ethics Committee of Sichuan University, China, No. KS2022984 (for detailed information of the submission to Ethics Committee, view)^1^. The customer service staff signed the agreement to participate in this study, allowing the research team to record both their physical and psychological emotional data, and authorizing the research team to use their personal information in academic papers after proper concealment, but not allowing for any type of commercial utilization of their information.

## Emotional data collection and pre-processing

We collected the customer services staff’s emotional data in the workplace. We chose the customer service staff from a large decoration company’s call center, headquartered in Chengdu, China. The company’s call center has two types of service mode: pre-sales and after-sales, which covers most types of emotional situations that exist during the service process between the customer service staff and customer.

### Sample

We recruited 29 full-time participants for our study. On average, the customer service staff were 27.556 (SD = 4.853) years old and had worked at this company for 2.211 years (SD = 2.266). Among the participants, 79.31% and 20.69% of the customer service staff identified as female and male respectively. About four-fifth (86.21%) of the employees held nonsupervisory positions and had at least a college degree (93.11%).

### Procedure

#### Data collection

**FIGURE 2:**
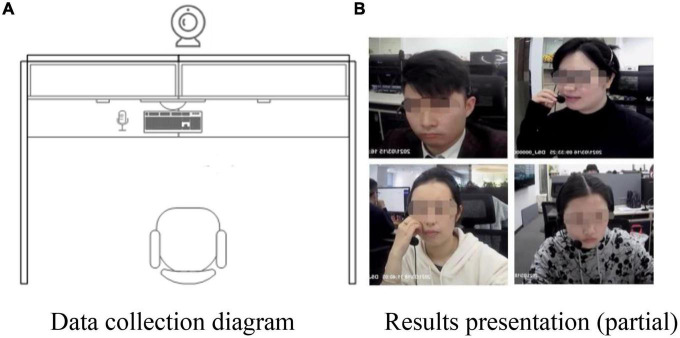
Data acquisition schematic. **(A)** Data collection diagram. **(B)** Results presentation (partial).

Our data acquisition method was to set up wide-angle cameras (device type: Aigo DSJ-T5) on the customer service staff’s workstations (see Figure 2A). The customer service staff were informed by their supervisor that they needed to cooperate with the collection of their emotional states for two weeks (from 9:00 am to 18:00 pm every day from March 15 to March 31, in 2021). The schematic of data collection is shown in Figure 2.

During the data acquisition process, in order to eliminate the resistance of employees as much as possible, the research team’s members promised each customer service staff member that their original emotional data would not be directly submitted to the company, and never be used to evaluate their job performance. More importantly, this research would not have any negative influence on their vocational development in the company. After data collection, the research team gave generous remuneration to the employees who successfully finished the experiment.

#### Data pre-processing

In order to explore the genuine emotional state of customer service staff, each phone call was set as a research unit in this research (Rueff-Lopes et al., 2015), and a pre-processing of the original video data was conducted. The standards of pre-processing were as follows:

(a) Keep the phone call records ∈ [0.5,5] miniutes;
(b) Intercepted manually from 15 seconds before the call was answered to 15 seconds after the call was hung up.

The reasons were that if the calling time was too short (0–30 s), the phone call usually contained limited information. Calls of this short time were normally hung up by the customers. However, if the calling time was too long (>5 min), it suggested that the customer was interested in the company’s business service, and the emotion of customer service staff was usually positive. At the same time, in regard to employees’ emotional labor, Rueff-Lopes et al. (2015) pointed out that customer service staff would unconsciously show their real emotions about the customer before and after the phone calls. Therefore, ±15 s calls were reserved to reflect the genuine emotions expressed by the customer service staff.

#### Results

We collected close to 10,240 GB of raw data. During the data collection process, two employees showed subjectively confrontational behavior, and another two employees resigned from this company for personal reasons. Thus, in order to ensure continuity of data, we excluded the relevant data of these four employees in the data analysis process. In all, the total amount of original data was about 9,215 GB (approximately 89.99% of the raw data). Of the eligible 25 participants, 19 were female (76%). The participants’ average age was 27.556 (SD = 4.853) years old and average organizational tenure was about 2.214 years (SD = 5.566).

To avoid the influence of disturbing elements, the acquired data were transcoded and unified by the Adobe Premiere Pro 2021. The resolution of data was adjusted to 960*540; the frame rate of the picture was adjusted to 30 frames; the sampling frequency of sound was adjusted to 48.0 kHz; all the data was saved as MP4 files.

The total duration of calls within 15 days was calculated as 154 h. Rueff-Lopes et al. (2015) had two trained researchers listen randomly to 967 live phone calls to narrow down the research material from 8,747 calls. Learning from Rueff’s method, 561 call records were selected by research team members as experimental samples from the original data. The data pre-processing results are shown in Table 1.

**TABLE 1:** Emotional dimensions for 561 phone call records.

Valence	Frequency	Ratio (%)	Arousal	Frequency	Ratio (%)
Negative	250	44.56	Low	153	27.27
Neutral	134	23.887	Medium	343	61.14
Positive	177	31.55	High	65	11.59

#### Discussion

This section provides solid data for our further research. Traditional studies are mainly based on external stimulation and arousal of the subjects’ emotion, rather than unconscious emotional expression. Through the collection of customer service emotional data in the workplace, our research can circumvent the inherent shortcomings of previous research methods (Du and Fan, 2007; Liu and Li, 2017), improving the authenticity and reliability of the results.

As can be seen from the emotional valence in Table 1, the amount of negative emotions is much greater than neutral and positive emotions. This indicate that employees are more susceptible to customers’ negative emotions in their service delivery and thus show emotional convergence. In terms of emotional arousal, service staff are less likely to show over excited or excessively negative emotions due to the objective conditions in the workplace (e.g., workstation environment, company’s rules and regulations, etc.). The results in Table 1 are consistent with actual situation.

## Study 1: Free association test of customer service staff’s emotional representations

In Study **1**, two groups of doctoral experts were recruited to engage in two consecutive tasks. Through a free association test (Lei et al., 2019), we provided a relatively comprehensive word pool of emotions for image selection *(Task 1)*, which then helped us construct a scientific scoring criteria/tool for subsequent research *(Task 2)*.

### Participants

We recruited 20 Ph.D. students who came from business school and school of electronics and information engineering, Sichuan University for our scoring criteria research. Participants’ average age was 26.2 (SD = 0.618). They all had normal or corrected-to-normal vision, and were right-handed. Fifty percent of participants’ research fields were identified as computer image emotion recognition, and the remaining individual research fields were organizational behavior and psychology. Further, there were differences in students’ research mindset for computer emotional recognition and organizational behavior and psychology, which provided reasonable scientific suggestions on the construction of scoring standards from different areas of study. All participants recruited had a minimum of two years’ research experience in emotional recognition. These two groups are abbreviated as: Expert 1: OB&Psy and Expert 2: CE&Rec.

### Procedure

**FIGURE 3:**
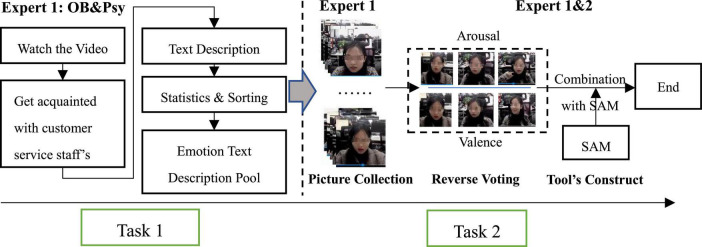
The flowchart of scoring criteria construction.

Study 1 consisted of two consecutive tasks (See Figure 3). ***Task 1:*** establishing a word pool of emotional representations. The members from Expert 1: OB&Psy, based on their understanding of each customer service staff during the collection data, were asked to write as many keywords as possible to properly describe the emotional state of each customer service staff. First, it was explained to Expert 1: OB&Psy that valence reflects the positive or negative degree of emotional level (negative, neutral, positive), and arousal describes the level of activation of body functions (low, medium, high) (Perveen et al., 2020). Emotions like joy, happiness, etc. can all be classified as positive, while, fear, anger, etc. are negative. Second, considering the normative and general characteristics of the written language (Olohan, 2004; Xu, 2014), Expert 1: OB&Psy were required to describe the emotional state of the customer service staff, using nouns or colloquial language as much as possible (Lei et al., 2019). Finally, the description of emotional range must include all three emotional states under the two emotional dimensions, namely: valence (negative, neutral, positive) and arousal (low, medium, high). The research team then counted the high-frequency words in each emotional state.

***Task 2:*** constructing a picture-type scale of the emotion of each customer service staff. Given the attribute of abstraction in written language, this task combined the SAM self-report scale to construct a picture scale to assist in displaying the emotional states of the customer service staff (Bradley and Lang, 1994; Bartosova et al., 2019). First of all, in the selection of pictures, we refer to previous picture selection criteria (VanHooff et al., 2013; Lei et al., 2019); that is, the pictures must have a clear theme, specific meaning, and can be understand directly without too much cognitive processing. Then, considering the emotional states of each customer service in the workplace, the members of Expert 1: OB&Psy were asked to find representative pictures (about 5–10 pictures/person) in the database (see Table 1). Third, Expert 1: OB&Psy and Expert2: CE&Rec were invited to complete a reverse vote about the picture’s intra-group consistency. That is to say, the more votes a picture got, the more it is significantly different from other pictures in the same emotional interval (valence or arousal). This picture would then be eliminated. Finally, considering that valence and arousal are different emotional dimensions, only three pictures were kept as a reminder for each customer service staff’s emotional state (see Figure 3).

### Results

#### Task 1

The statistical results of verbal descriptions of customer service staff’s emotional states in different emotional situations are listed in Table 2. In the classification of the two fundamental dimensions of emotions: Valence (negative, neutral, positive) & Arousal (low, medium, high), 9-Likert scales were used to distinguish scores interval. Specifically, Negative & Low∈[1,3], Neutral& Medium∈(3,7], Positive& High∈(7,9].

**TABLE 2:** The text classification of emotional valence & arousal.

Emotional dimensions	Categories & score’s interval	Text presentation
Valence	Negative∈[1,3]	Frowning, tightly closing eyes, lips pressed together, eyes rolled back…… (FE); sighing, swearing, speaking faster……(CV); body swinging from one side to the other, supporting the head with the hands……(BL)
	Neutral∈(3,7]	Calm in facial expression……(FE); moderate voice speed……(CV); upright posture……(BL)
	Positive∈(7,9]	Smiling or giggling, laughing out loud……(FE); playfully chatting with laughter……(CV); body shaking, nodding……(BL)
Arousal	Low∈[1,3]	The emotional state of customer service staff is very low and activity is at the lowest level
	Medium∈(3,7]	The emotional state of customer service staff is calm and activity is at a normal level
	High∈(7,9]	The emotional state of customer service staff is very excited and activity is at the peak

##### Emotional valence

In the classification and induction of the text written by Expert 1: OB&Psy, we mainly summarized the word groups into three aspects: body language (BL), voice communication (VC), and facial expression (FE) (Baltrušaitis et al., 2018; Lackovi, 2018; Ethriaj and Isaac, 2019; Wang et al., 2019). Then, we counted the frequency of the vocabulary description of valence, based on the words written by the Expert 1: OB&Psy group. Then, we formed the textual presentation about the customer service staff under each category & score interval.

##### Emotional arousal

In order to count emotional arousal and to distinguish the meaning of arousal and valence, we made an abstract representation of arousal from the level of vitality and energy. Low arousal refers to “the emotional state of customer service staff is very low and activity is minimal”. Medium arousal means that “the emotional state of customer service staff is calm and activity is at a normal level”. High arousal denotes that “the emotional state of customer service staff is very excited and activity seems to be at the peak”.

#### Task 2

**FIGURE 4:**
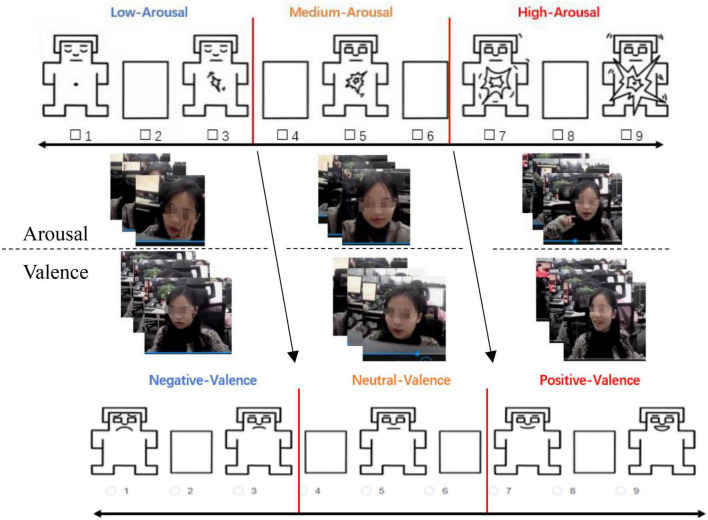
Examples of customer-service staff’s auxiliary emotional judgment scale.

**Notes:** the valence and arousal as two dimensions of emotional, it often easy to misunderstand each other, therefore, we first evaluate the valence, and after a period of time, the arousal were be evaluated.

Since verbal scales are more sophisticated and arcane than picture scales, it’s more difficult for research subjects to evaluate emotions using the former one. Hence, we adopted the method of reverse voting and combined the SAM scales to construct a set of picture-type scales. Under the 2 × 3 emotion combination (two dimensions and three categories), we constructed an auxiliary emotional judgment scale, including 18 pictures for each customer service staff, shown in Figure 4.

### Discussion

The outcomes of Study 1 provide scientific scoring criteria and an auxiliary tool for our further emotion labeling. Our scoring criteria (see in Table 2) provides a good guidance for subsequent research evaluating the emotional state of customer service staff. Also, our method can avoid the emotional recognition error and common method bias caused by evaluator’s cognition and social experience (Yao et al., 2018; Jordan and Troth, 2019). Further, the picture-type scale is relatively concise and intuitive (Gueldner et al., 2005; Charlotte et al., 2020), which can help first time raters quickly form a judgment on how customer service staff exhibit and express their emotions in the workplace.

## Study 2: Evaluation of emotional states in the workplace

In Study **2**, Expert 1: OB&Psy and Expert2: CE&Rec evaluated the emotional information of the customer service staff in 561 phone call records (see Table 1). The picture-type scale integrated with SAM that we developed in Study 2 was used. To decrease the scoring error caused by job burnout (Schabram and Tseheng, 2022), the average experiment time were from 9:00 a.m. to 11 a.m in one week. Specifically, Expert 1: OB&Psy evaluated the emotional states in the first three days; their results were compared with the original data (see Table 1) and the consistent ones were kept (Adolph and Alpers, 2010). Expert2: CE&Rec participated in the experiment from the 3rd day to the 6th day, and the 7th day was held as supplementary experiments for those who were unable to participate in the experiments on time. In addition, in order to maximize the quality of the scoring results, the students was promised that they could receive a generous reward of ¥ 300 per day. After the experiment, if a PhD expert’s score was within ± 1 standard deviation compared to the sample mean of the whole group, then a bonus of $100/day would be paid.

### Participants

The participants in Study 2 were fully consistent with those in Study 1. We continued to utilize the two groups of PhD students (Expert 1: OB&Psy and Expert2: CE&Rec) because they not only had differences in research mindset and logic but also had a comprehensive understanding of the emotional expression rules of the customer service staff after attending Study 1. To increase the accuracy of the evaluation results, the two groups of doctoral students were organized to conduct experiments independently in a classroom that had the same lighting conditions, computer resolution (1,600 × 1,024), and noninterference features as before.

### Procedure

**FIGURE 5:**
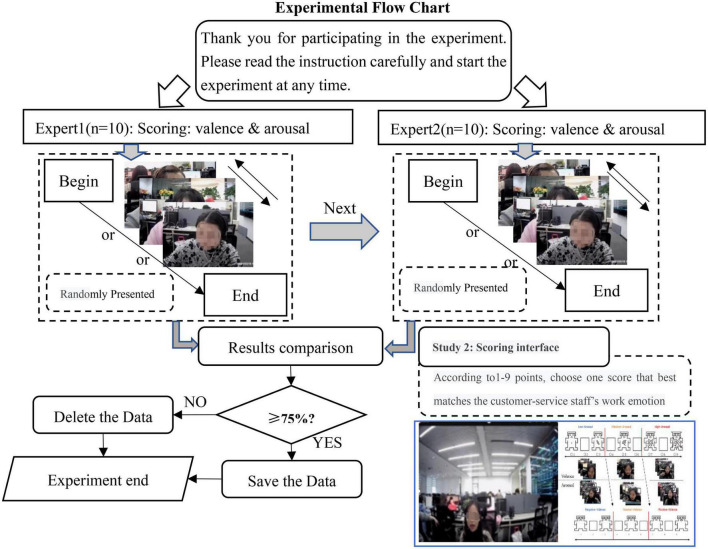
Experimental flow chart of Expert 1 & 2.

In Study 2, our purpose was to annotate the emotional information for the 561 phone call records (the operation flow chart can be seen in Figure 5).

First, we explained some important notions to each participant, such as the meaning of valence and arousal, and that the object of the evaluation was the customer service staff’s work emotions rather than the emotions of participants themselves as motivated by the customer service staff. Then, Expert 1: OB&Psy and Expert 2: CE&Rec were asked to label the emotional state of each customer service staff independently. We uniformly used the QQ Player to randomly present the work states of each employee. When scoring each phone call record, participants were asked to label emotion through a “category first, scores next” approach. Taking valence as an example, participants need to determine the range (negative or neutral or positive) of the customer service staff’s emotion, and then compare the schematic diagrams to decide an appropriate score: if a phone call record was determined to be in the negative range, participants could score as 2; if the degree was very high, 1, otherwise 3.

To improve the accuracy of emotion labeling and avoid the risk of misjudgment, we also asked each participant to briefly write the reasons for their scoring after each data annotation was completed. Adolph and Alpers (2010) believes that when the intra-group consensus rate is greater than 75%, the accuracy of emotion judgment improves. Therefore, we set the consensus level to be ≥75% and retained the data that accorded with the scoring results.

### Results

Tables 3, 4 show our research results. From the statistical results of the data (Table 3), it can be seen that, under the constraint of consistency ≥75%, the average scores of 10 students in the Expert 1: OB&Psy group are consistent with the original data’s emotional categories. Based on this result, the emotion labels show a tendency to deviate when adding the results that were evaluated by Expert2: CE&Rec. Finally, after two rounds of scoring, a total of 227 (40.46%) stable emotional data were obtained, which were named Stable 0 (used in Study 4). Among them, the distribution of emotion categories was 73 negative, 94 neutral and 60 positive.

**TABLE 3:** Descriptive statistics of evaluation results.

	Negative	Neutral	Positive	Total	Ratio (%)
Original data	250	134	177	561	–
Expert1:OB&Psy	93	162	44	299	53.29%
Expert2:CE&Rec	101	156	42	299	–
Results (≥75%)	73	126	28	227	40.46%

**TABLE 4:** Consistency between Expert 1 and Expert 2 in judging emotional valence & arousal (≥75%).

Emotional dimensions	Categories	Minimum	Maximum	Mean	SD	*N*
Valence	Negative	1.583	3.000	2.421	0.086	73
	Neutral	3.001	6.993	5.284	1.066	126
	Positive	7.000	7.892	7.331	0.063	28
Arousal	Low	2.000	3.000	2.683	0.058	62
	Middle	3.240	6.560	5.018	0.556	139
	High	6.000	8.545	6.438	0.269	26

As shown in Table 4, our research results contain two emotional dimensions: valence and arousal. The statistical results of the valence and arousal show that the emotional score basically covers all emotional types in the workplace. In the score of the valence dimension, the mean score of negative, neutral and positive emotions are 2.421 (±0.086), 5.284 (±1.066) and 2.421 (±0.086) respectively. The results of emotional arousal under the three categories are 2.683 (±0.058), 5.018 (±0.556), and 6.438 (±0.269). This illustrates that the data screening and pre-processing method in Study 1 has great practicability (Rueff-Lopes et al., 2015). In addition, in terms of the volume of data, the largest one is the neutral category (126). The negative ones (73) are much more than the positive ones (28). This implies that negative emotions occur in the workplace far more frequently than positive emotions, which is consistent with the actual situation (Hatfield et al., 1993; Sykes, 2015; Liu et al., 2019; Perveen et al., 2020). Further, it indirectly shows that effectively identifying and managing workplace emotions has important practical significance.

### Discussion

To evaluate the customer service staff’s work emotion in each phone call record (in Table 1), in Study 2, we used a new scoring criteria and evaluation tool that we constructed in Study 2. Two groups of PhD candidates participated in our experiments. First (see Table 3), we found that only 40.46% of the emotional labels remained stable from the simple classification of the original data to the determination of the final label. At the same time, comparing the results of the scores within the expert groups, we also found that the volume of negative data increased to 101 from the original 93, while neutral and positive data decreased. This suggests that emotion is a very complex system, and people’s understanding and final judgment of the emotional state of objects are also largely affected by cognition and social experience (Camacho et al., 1991; Liu et al., 2019).

Second, we found almost no extreme emotional states (i.e., scoring outcomes is 1 or 9), either in the dimension of valence nor arousal. This could be attributable to the emotional self-regulation of the customer service staff and corresponding emotional adjustment tactics in the organization. Research has shown that when an employee’s work continuously leads to service failure (Gronroos, 1988; Hazée et al., 2017; Liu et al., 2019), whether it is attributed to subjective (Bowen, 1999) or objective (Hazée et al., 2017) factors, the employee will take actions to suspend his/her work for some time, during which time the employee is required to adjust their emotions.

### Supplemental analysis

Study 2 provided a set of stable scientific emotional data for our study. However, Study 2’s results did not give a good explanation of why nearly 60% of the original data deviated when the two groups of scoring process ended (see Table 3). The answer to this question may be found in the way the experts labeled the emotional states (see Table 5).

**TABLE 5:** Emotional evaluation of the experts and presentation of the reasons.

Data number		Expert A	Expert B	Expert C	……	Reserved?
01	**Labels**	**Positive**	**Positive**	**Positive**	……	Yes
	Reason statement	Seems happy during the phone call	Amused by a joyful and humorous customer	Laughing with colleagues afterward		
02	**Labels**	**Negative**	**Negative**	**Negative**	……	Yes
	Reason statement	Gradually became grumpy because the customer was whiny and unhappy in the phone	Impatient, pressing the lips together	Voice became louder, and finally there was the action of smashing the phone		
03	**Labels**	**Negative**	**Negative**	**Negative**	……	Yes
	Reason statement	The signal quality of the phone call is not good, the employee complains later	Complaining with coworkers after hanging up	Complained with their colleagues		
04	**Labels**	**Negative**	**Neutral**	**Negative**	……	No
	Reason statement	Dispute on the telephone	Facial expression is normal	Repeatedly explaining to the customer, and rolling his eyes		
……	……	……	……	……	……	……

As shown in Table 5, when scoring the emotions of the same data, even if the results are the same, the focus of each expert is different. For instance, in data numbered 02, expert A paid attention to the communication content between customer and employee, while expert C was more influenced by the actions of the customer service staff. On the other hand, Study 3 was essentially conducted by a “self-report” method (Bradley et al., 2001; Li et al., 2005; Kuesten et al., 2014), which required each expert to be able and willing to report their feelings. However, the actual results show that even when participants are willing to report their feelings, the focus of attention is not the same. Therefore, the emotion measurement method based on “self-report” has a greater risk of distortion in practical application.

In addition, when summarizing the reasons for scoring the 227 pieces of data, we found that the experts focused on three key elements to determine the emotional states of the customer service staff: facial expression, body language and voice communication. In the process of analyzing the reasons for the scores, we mainly used the coding method of qualitative research to briefly classify the text descriptions from experts (Strauss, 1987).

**FIGURE 6:**
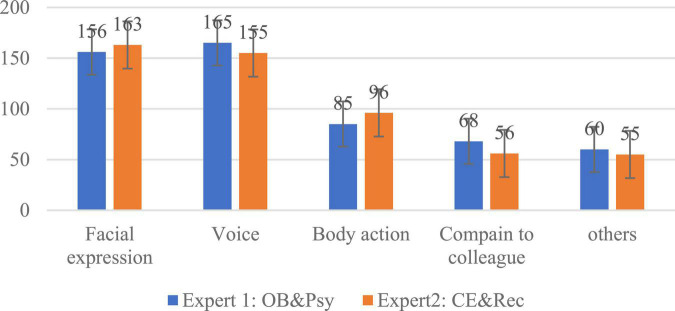
Statistics of high-frequency reasons for judgment of emotions.

For instance, text descriptions such as “complaining with colleagues,” “is amused,” and “dispute on the telephone” were attributed as voice communication; “smashing the phone” and others were coded as body language, while “rolling eyes back,” “pressing their lips together” etc. were coded as facial expression. Finally, we sorted out and coded about 56,700 Chinese words written by 20 experts, and plotted the high-frequency reasons that affected emotional judgment, as shown in Figure 6.

As can be seen in Figure 6, facial expressions, voices, and body language are the three most important modalities the expert groups identified to judge customer service staff’s emotions. In the two expert groups, these three factors accounted for 76.29 and 78.85%, respectively. At the same time, situational factors in the workplace, such as complaining with colleagues, had a significant influence on emotional scores. The proportion of other factors only accounted for 11.23 and 10.66%. We wonder, what is the role of these factors in the process of transmitting emotional information? Are there differences between each factor? And through what channels does the influence happen? Clarifying these issues is important for effectively judging and managing emotions in the workplace.

Last but not least, in the process of listening to each audio of call center staff, we found that customer service staff tend to unconsciously imitate the emotional state of the customer. For example, when encountered with a bad-tempered customer, the call center employee will also be easier to get irritated. On the contrary, if the customer is joyful and in a good mood, call center employee will be more patient and tender. This phenomenon proved that emotional contagion exists during the interaction between customers and call center employees.

To sum up, based on the above problems, we define the scoring results of Study 3 as “Stable 0,” meaning a standard result of theoretical analysis which were used in the next study.

## Study 3: Evaluation of emotional states based on the multimodal dataset

In Study 3, we constructed a multimodal dataset based on the multimodal emotion recognition method and the scoring attribution results (see Figure 6). The duration of the experiment was three weeks, and the specific times each day were consistent with Study 3.

### Participants

We recruited 76 volunteers from online to participate in this experiment, because social samples may be more likely to use practical and open-minded approaches to evaluating emotions. To motivate participation, we paid ¥ 150 as a reward to volunteers who successfully completed all three modalities’ evaluation. At the end of the experiment, 12 volunteers failed to finish the entire process due to personal reasons. The emotional data submitted by 4 volunteers were missing. Therefore, a total of 60 volunteers successfully participated in Study 3, with a participant rate of 78.95%. Among the 60 subjects, 32 (53.33%) were male and the others (46.67%) were female. On average, participants were 22.38 (SD = 2.914) years old; 46.67 and 53.33% of them had a university degree in natural science or social science respectively.

### Procedure

**FIGURE 7:**
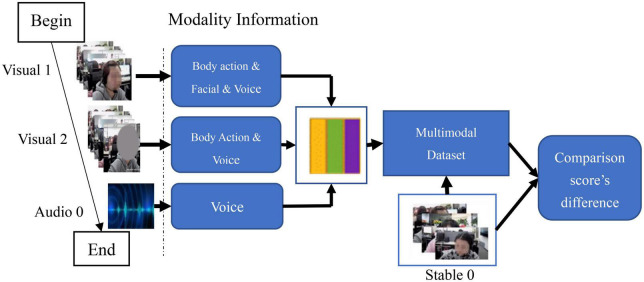
Scoring flowchart for multimodal dataset of volunteers.

There were two consecutive tasks in Study 3 (see Figure 7): ***Task 1***: multimodal dataset construction; ***Task 2***: volunteers’ scoring. Before the tasks began, all volunteers were organized to take a course explaining vital concepts and procedure of the experiment.

In ***Task 1***, we completed the construction of a multimodal database based on the conclusion of Study 2 that facial expression, voice, and body language were the three key elements that induced the experts to make final emotional scores. We also explored how these three elements affect experts’ judgments and carry emotional information.

First, we dismantled the Stable 0 dataset using Adobe Premiere Pro 2021, and sorted it out into two human sensory channels: visual and auditory (Lahat et al., 2015; Yoon et al., 2015; Wang et al., 2019). The visual channel refers to the emotional information conveyed by facial expression and body language in Stable 0. The auditory channel refers to the voice communication between customer and employees.

Second, we transformed the information into two modalities: Visual 1 (containing body language & facial expression & voice) and Visual 2 (containing body language & voice). We also downloaded the corresponding telephone records from the company’s Cloud database, labeled as Audio 0, to complete the customers’ voice data missing in Stable 0. In addition, considering that the proportion of body movements is comparable to situational factors (e.g., complaining with colleague, see Figure 6), and there are difficulties in extracting modality information, we set body language as a control variable in the setting of the multimodal database. The composition and operation method of the multimodal emotional database is shown in Table 6.

**TABLE 6:** Composition and interpretation of multimodal emotional database.

Label	Major modality information	Data sources and extraction methods
Stable 0	Body language + voice + facial expression	Containing full information on the employees’ emotions in the workplace, including facial expression, posture, phonetics and intonation, communication with colleagues, etc.
Visual 1	Body language + facial expression	Taking ‘phonetics and intonation’ out of Stable 0
Visual 2	Body language + voice	Taking ‘facial expression’ out of Stable 0.
Audio 0	Voice information between employee and customer	Voice-to-voice communication between customer and employee, which was downloaded from the company’s Cloud database.

In ***Task 2***, we completed the scoring on the multimodal emotional database. Over 3 consecutive weeks, volunteers submitted emotional scoring results for 3 modalities (Visual 1, Visual 2 and Audio 0). Given that each sub-data set was derived from another set, the experiments were arranged weekly to minimize experience and learning effects (Nomura et al., 2009). Specifically, the scoring of Visual 1 and Audio 0 were completed in the first and second weeks respectively, and Visual 2 was scheduled to be completed in the third week (Monday to Friday). The weekends (Saturday and Sunday) were used for supplementary data collection. The reason for the above evaluation sequence is that dynamic pictures (Visual 1) have a weaker effect on the rater (Guedes et al., 2022) than voice stimuli (Audio 0). More importantly, our research used heavy mosaic (see Visual 2, in Figure 6) to cover the facial expression, which made it difficult for raters to associate Visual 1 with Visual 2 during the rating process.

Finally, as in Study 3, volunteers were placed in a quiet and uncluttered classroom had consistent lighting intensity to conduct the experiment. The computer resolution was set to 1,600 × 1,024 uniformly. Volunteers were asked to fill in the emotional valence & arousal scores of customer service staff in three modalities, and briefly explain the reasons.

### Results

#### Task 1: Multimodal dataset construction

Study 2 provided us with 227 stable emotional data (see Table 4). Based on these results, we constructed a multimodal dataset. During the process, we mainly faced two problems: (1) After the phone call records were downloaded from the Cloud, some of the data were difficult to match with the data collected by the wide-angle camera, because the volume of information transmitted by the voice was too small; (2) When providing subsequent services to customers (both pre-sale and after-sale service), some employees tended to use personal mobile phones to complete the call, which caused missing records in the Cloud database. Due to these two issues, we only successfully constructed 99 (out of 297) sets of multimodal data for study 3.

#### Task 2: Volunteers’ scoring results

In each modality, the mean values of emotional valence & arousal are shown in Table 7. We found that the inter-class correlation coefficients of each modality were significant (*ICC*_*min*_ = 0.733, *P* < 0.001), whether the valence or arousal dimension of data. The minimum value of Cronbach’s αfor each sub-modality was 0.733 (*P*<0.001), indicating significant multimodal effects of the database.

**TABLE 7:** Evaluation results of multimodal database of valence & arousal (volunteers).

Emotional dimensions	Label	Valence			Arousal		
		Mean ± SD	ICC	α	Mean ± SD	ICC	α
Negative & low	Stable 0	2.478 ± 0.084	–	–	2.662 ± 0.056	–	–
	Visual 1	4.274 ± 0.399	0.733	0.724	4.277 ± 0.292	0.842	0.833
	Visual 2	3.484 ± 0.388			4.728 ± 0.158		
	Audio 0	5.061 ± 0.239			5.014 ± 0.323		
Neutral & medium	Stable 0	4.853 ± 0.186	–	–	4.641 ± 0.408	–	–
	Visual 1	5.425 ± 0.151	0.887	0.854	4.924 ± 0.454	0.865	0.865
	Visual 2	4.916 ± 0.265			5.347 ± 0.407		
	Audio 0	5.259 ± 0.186			5.043 ± 0.406		
Positive & high	Stable 0	7.059 ± 0.081	–	–	7.072 ± 0.104	–	–
	Visual 1	5.667 ± 0.385	0.764	0.899	6.304 ± 0.234	0.756	0.798
	Visual 2	6.275 ± 0.165			5.610 ± 0.202		
	Audio 0	5.275 ± 0.099			4.907 ± 0.258		

**FIGURE 8:**
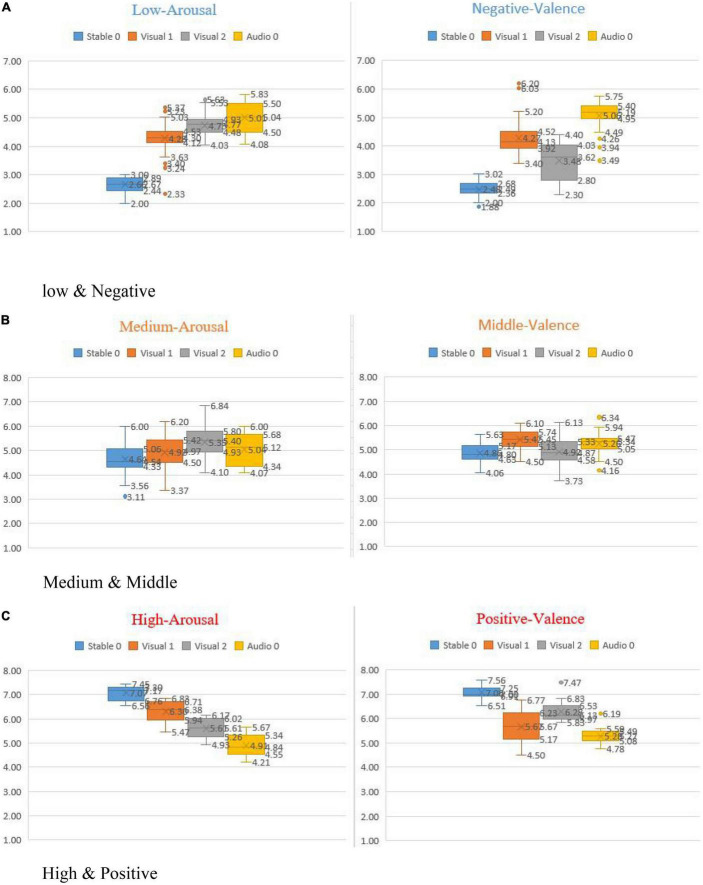
Box plots of emotion scores of multimodal database. **(A)** Low and negative. **(B)** Medium and middle. **(C)** High and positive.

As shown in Table 7, we found that, compared with the sample mean of Stable 0, the degree of deviation of three sub-modalities in the negative-valence showed the following trend: Audio 0 (M = 5.061, SD = 0.239) > Visual 1 (M = 4.274, SD = 0.399) > Visual 2 (M = 3.484, SD = 0.388) (see Figure 7). In addition, there was a similar tendency in the dimension of low-arousal, that is, Visual 2 (M = 5.610, SD = 0.202) > Visual 1 (M = 6.304, SD = 0.234). More importantly, in other emotional dimensions (Neutral & Medium and Positive & High), this pattern remained consistent (Figure 8).

The paired sample t-test for valence shows that there was no significant difference in the sample mean between positive (*t* = 4.067, *p* = 0.0000) and negative (*t* = 4.736, *P* = 0.001), when controlling for the body language of the customer service staff. The original positive data and the negative data were the closest to the sub-modal Visual 2 (M_stable0,positive_ = 7.059 ± 0.081 & M_visual2,positive_ = 6.275 ± 0.165; M_stable0,positive_ = 7.059 ± 0.081 & M_stable0,negative_ = 2.478 ± 0.084). This implies that when facial expression conflicts with voice (compare the explanations of Visual 1 and Visual 2, see Table 6), the voice dominates the raters’ judgment of the emotion valence of customer service staff. On the contrary, the results of negative (4.274 ± 0.399) and positive (5.667 ± 0.385) in Visual 1 were located at the neutral level, indicating that facial expression did not play a dominant role in the judgment of extreme emotions.

Similarly, a paired sample t-test of the emotional arousal showed that the sample mean values between low (*t* = 4.494, *p* = 0.000) and high arousal (*t* = 3.392, *p* = 0.001) exhibited no significance. The original low and high arousal data were the closest to the sub-modal Visual 1 (*M*_*stable* 0, *low*_ = 2.662 ± 0.056 & *M*_*stable* 0, *high*_ = 7.072 ± 0.104). Analogously, this shows that when facial expression conflicted with voice, facial expression dominates the volunteers’ judgment of emotion arousal states. Further, volunteers can judge the emotional state (low, medium, or high) of customer service staff more accurately through facial expressions.

To sum up, the findings of Study 3 suggest that facial expression and voice play different roles in judging the emotional states of customer service staff in the workplace. Among them, voice (phonetics and intonation) is the key element to convey customer service staff’s emotional valence (positive, neutral, negative), while facial expression mainly reflects emotional arousal (low, middle, high).

### Discussion

To extend our findings in Study 2, in Study 3, we constructed a multimodal database to explore the mechanism behind the reasons for the emotional judgments of Expert 1 & 2 groups. Two conditions that affect the judgment of the emotions were discovered in Study 4. First, we found, through comparison of sub-multimodal datasets, that facial expression and voice play different roles in conveying true emotions. This suggests that front-line managers can use methods to monitor employees’ voice and facial expression to regulate and control negative emotions in the workplace. Second, we found that the scoring results of Audio 0 were only at neutral and middle level, whether in valence and arousal. This could be attributable to employees’ emotional labor (Destephe et al., 2015). Under the constraint of organizational rules, employees cannot directly release their negative emotions through the phone calls, and they can only express them through other methods, which may be unconscious to some extent (Bauch et al., 2015; Rueff-Lopes et al., 2015; Liu et al., 2019).

Third, Study 3 provided empirical evidence for how to judge and manage the emotions in the workplace: whether customer service staff are happy (valence) or activated (arousal) can be identified by voice and facial expression respectively. Moreover, in constructing the multimodal database, we set the body language as a control variable because the work stations’ space is limited. Meanwhile, the Expert’s reasons for scoring demonstrate the non-relevance of bodily actions in judging emotions in the workplace. Therefore, this shows that, compared to the judgment of athletes’ emotions on the sports field (Aviezer et al., 2012; Wang et al., 2021), the judgment of emotions based on emotional modality cues should be considered in combination with specific situations.

## General discussion

This article was inspired by some contemporary phenomena in China: the application of 5G is increasing the profitability of online services (Khan and Zhang, 2007; He et al., 2013) and the negative externalities brought by the COVID-19 pandemic (Wheaton et al., 2021). Employees may bring negative emotions with them to work, causing losses to the company. Considering the complexity of emotions, we applied a multimodal emotion recognition method to judge effectively how customer service staff expressed their emotions toward each phone call; our aim was to summarize the rules of expression of workplace emotions. In this way, we provide insights into how to judge the emotions in the workplace.

Across three consecutive studies, customer service staff usually act out the work emotions needed by the organization through voice, which is usually known as emotional labor. However, their real emotions were often unconsciously exhibited via facial expressions and bodily action. This shows that single-modal data alone can easily lead to distorted judgments, so multimodal emotion recognition analysis is of great necessity. We also noted in Study 1 that both groups of experts were affected by similar factors such as facial expression (FE), voice communication (VC), and body language (BL) in judging a certain emotion (see Table 2), even when evaluating different objects. However, the words used to describe the emotional states by different expert were different from each other. Taken together, these phenomena imply that representation and judgment of emotions in real situations should rely on a concise and intuitive picture-type scale rather than a traditional text-type scale.

We also found in Study 2 that each rater’s judgment of the final emotional scores was affected by different factors. Even when the rating processing was sequential (Expert 1: OB&Psy first, then Expert2: CE&Rec), the consistency of results was only at the level of 40.46% (See Table 3). That is to say, even when the final labels of emotional state were in the same range, the focus of each expert was very different (See Table 5). In Study 3, based on the high-frequency encoding results (See Figure 6), we constructed a multimodal dataset. We unexpectedly found that, by comparing the degree of deviation of each sub-modality with Stable 0, emotional valence and arousal play different roles when a third-party evaluates the emotional states. In other words, when judging workplace emotions, valence showed a good fit with employees’ voice, and arousal was mostly associated with facial expression.

Finally, it is worth mentioning that the results of this article emerged from a real workplace and a series of contextual emotion analysis experiments (Aviezer et al., 2012). We achieved the objectives of this research across three sub-studies: collection of emotional data from 29 customer service staff (in Study 1); a scoring criterion and an auxiliary tool based on a picture-type scale (in Study 1); and two scoring experiments. The participants of our studies were from three populations. This research design made sure that the results were generated from practices and that the research was not an “imprecise replication” (Schabram and Tseheng, 2022) about different participants, but rather a stable result via testing different participants. We realized that the judgment of emotions can be largely influenced by contextual factors (e.g., Wang et al. (2021) and Aviezer et al. (2012). Therefore, further studies should focus on integrating experiment with practice.

## Theoretical implications

This article makes some important contributions to theoretical research. First, we contribute to the emotional management literature by evaluating customer service staff’s work emotions in a real situation. Most research has recognized the importance of judging and managing emotions in the workplace (Michel, 2001; Du and Fan, 2007), but the methods for studying emotions depend largely on external stimulus and self-reported scales (Bradley et al., 2001; Li et al., 2005; Kuesten et al., 2014). Yet, as we noted at the outset, the attributes of emotions include biological and socio-cultural influences. Research into work emotions requires consideration of the internal and external factors of work situations as well as the necessity for emotional interactions between employees and customers. Therefore, we collected emotional data that affected customer service staff’s work by using a wide-angle camera. Further, we use an experimental method to conduct our research (Aviezer et al., 2012). By recruiting two groups of experts and one group of volunteers, we summarized the main factors that affected the customer service staff in the workplace (see Table 5 and Figure 6).

Second, this research provides a set of ideas for constructing scientific auxiliary tools for evaluation of workplace emotions. As previously mentioned, the short duration and high intensity of emotions (Camacho et al., 1991; Ekman, 1994; Gao et al., 2019) make it difficult to report one’s real-time emotions with traditional text-type scales, such as PANAS, PAD, SDS, etc. (Bradley et al., 2001; Kuesten et al., 2014). Accordingly, we constructed scoring criteria and tools using a so-called “free association test” in Study 1. Lei et al. (2019) and VanHooff et al. (2013) believed that, compared to text, pictures have clear themes and more specific meaning and can be easier to understand without too much cognitive processing. Therefore, we combined pictures with SAM (Bradley et al., 2001) to construct an auxiliary emotional judgment scale about customer service staff’s emotional states (see Figure 4). By developing a picture-type scale, this work supports improvement of the accuracy of emotional judgment.

Third, this work applies the idea of multimodal emotional recognition to experimental research into the psychosocial and organizational field. Due to the diversity and complexity of emotions, emotional judgment based on a single-model can easily lead to the self-serving bias of impression management (Lahat et al., 2015; Yoon et al., 2015; Jordan and Troth, 2019; Wang et al., 2019). For instance, a sentence may contain multiple and conflicting emotions (Perveen et al., 2020), and positive language may veil sarcasm (Gao et al., 2019). Therefore, emotional judgment and recognition has gradually expanded from single-modal to multi-modal, becoming a social science and natural science research category (Wang et al., 2019). We apply this refinement to the emotional judgment of customer service staff in the real workplace. The practice of this theory not only conforms to the new trend of service industry transformation in the digital age, but also explores the characteristics and principles of customer service staff’s emotional representation in the voice-to-voice channel. Taken together, the successful application of the multimodal emotional recognition in this article provides a new theoretical and methodological approach for social science scholars to accurately judge and identity employees’ work emotions in the workplace.

Fourth, our work explores how each emotional modality conveys the emotions of customer service staff in the workplace. Specifically, when bodily actions were set as control variables, facial expression and voice were strongly associated with emotional arousal and valence respectively. In addition, under different levels of valence & arousal, customer service staff’s use of voice all scored at the “medium & neutral” level (see Table 6). This indicates that there clearly exists emotional labor when delivering service to customers, which is consistent with the actual situation. From a methodological point of view, our results were obtained by comparing the differences in sentiment scores across different modalities. To a certain extent, this research can improve the credibility of the results of traditional research where data is collected by questionnaires since we fixed the problem of self-serving bias through expert evaluation.

Finally, a total of 99 (out of 297) sets of high-quality multimodal datasets were constructed, aiming to achieve the goal of this article. In further research, this dataset can not only support psychological and behavior research, but also can be expanded to a “Chinese Customer Service Staff Affective Multimodal System”. This system will be able to provide scientific data support from real work situations for the development of computer emotion algorithms.

## Practical implications

In addition to the theoretical contributions of this research, we offer several practical implications. First, for employees. Effective emotional management is not to compensate for the various negative consequences brought by emotional disorders, but to work with a good state by self-regulating bad emotions. Once one decides to manage work emotions, one should pay attention to his/her emotional representation in three aspects: facial expression, bodily action and voice. On this basis, the situational factors of the workplace can affect employees’ emotional states (See Figure 6), such as communicating with colleagues (Rueff-Lopes et al., 2015). From the results of the attribution of emotion states (Tables 2, 5), the occurrence of specific events in the workplace can help to regulate the negative emotions that come from the customer (for instance, simple communication and complaining with colleagues after the phone call can help to soothe negative emotions). Thus, the diversion of attention can effectively help employees adjust their emotions when suffering from negative emotions.

Second, for managers, maintenance of good emotional states of employees can not only affect the working atmosphere, but also enable the team, department, and even the entire enterprise to achieve higher performance. Managers must be aware that in the service industry, whether in the long or short term, the emotions of employees can directly affect customer satisfaction, which will be reflected in fewer service complaints. Therefore, managers need to focus on how to judge the emotional states of employees. In practice, front-line managers can judge whether an employee is active or positive by observing their facial expression and voice respectively, and then rationally determine whether the employees’ emotions can support the next service. Finally, our research suggests that managers need to take “prevention first, intervention next” as a basic principle to manage emotions in the workplace. Faced with a potentially negative psychological problem in employees, mangers can regulate these problems through education, counseling with employees, and crisis intervention.

Third, for organizations, they must acknowledge that the traditional model of business service has changed under the influence of digital technology and Web 2.0. As the new generation of employees enter the workplace, they prefer a good organizational climate and a comfortable working environment. Therefore, organizations not only need to provide training suitable for the digital age to improve the employees’ service capabilities, but also need to support the ideological change in employees’ working ability and organizational identity under the new work mode.

## Limitations and directions for future research

The main purpose of this paper is to explore how to identify the work emotions of customer service staff in the real workplace, and to conduct a series of empirical analysis. Future studies can be carried out from the following aspects.

First, based on affective events theory (Weiss and Cropanzano, 1996), follow-up research can explore the influence of the emotional interaction between customer and employees during the process of online service delivery. In Study 3, we summarized the factors affecting how experts score the emotional states of customer service staff in the workplace. The results showed (see Figure 6) that contextual strategies such as employees complaining with colleagues need research attention. Communication and interaction in this digital age differ from patterns in the era of offline service: how does online service affect customer service staff’s emotions? Are there relatively stable mechanisms or general patterns? There are a lot of theoretical and practical issues to be explored in further research.

Second, our research has shown that facial expressions and bodily actions have different roles in conveying customer service staff’s emotions. We found a very interesting phenomenon in experts’ reasons for evaluating employees’ emotional states in Study 3: customer service staff tend to unconsciously imitate the emotional state of the customer. In other words, emotional contagion is very common in online services. There has been evidence indicating that the negative emotions that customers evoke in service staff are largely determined not by *what was said*, i.e., the content of conversations, but by *how they say it*, i.e., the attitudes of the speakers (Rueff-Lopes et al., 2015). Therefore, from the perspective of emotional contagion, further research is needed to explore the underlying reasons, manifestations, and mechanisms of emotional contagion between customers’ and employee’s emotional interaction in the voice-to-voice situation.

Third, future research can investigate the relationship between emotions and organizations. We have summarized the relevant characteristics of customer service staff emotional representation in the workplace. However, we found that emotional interactions between customers and employees will affect the emotional states of employees, which in turn determine their service behavior choices, and finally affect the company performance. According to this logic, we question what types of service modes employees should use and what the relationships between each type of service mode and the emotional responses of both customers and employees are. Finally, it’s worth researching whether the service modes have a linear relationship with corporate performance, that is, whether they have a direct effect on the outcomes of the organization. Exploring these problems will have a theoretical and practical significance for organizational performance management.

## Conclusion

Our research interrogates how to judge the emotional state of customer service staff in the workplace effectively. Strong evidence collected by a wide-angle camera was used to explore the customer service staff’s emotions in the real-life situation. This work offers ways of refining the tools for emotional measurement by providing score criteria and a picture-type scale (see Study 1). The study found strong evidence that voice (phonetics and intonation) is the key element to convey customer service staff emotional valence (positive, neutral, negative), and that facial expression mainly reflects emotional arousal (low, middle, high).

## Data availability statement

The raw data supporting the conclusions of this article will be made available by the authors, without undue reservation.

## Ethics statement

The studies involving human participants were reviewed and approved by Ethics Committee of Sichuan University, China, No. KS2022984. The patients/participants provided their written informed consent to participate in this study. Written informed consent was obtained from the individual(s), and minor(s)’ legal guardian/next of kin, for the publication of any potentially identifiable images or data included in this article.

## Author contributions

PL and YZ were responsible for writing the manuscript. ZX and LQ were in charge of the design of experiment and the development of database. YW was responsible for the revision and typesetting of the manuscript. All authors contributed to the article and approved the submitted version.

## Footnotes

1
https://wcums.scu.edu.cn/info/1078/2802.htm
